# Adolescents’ loneliness in European schools: a multilevel exploration of school environment and individual factors

**DOI:** 10.1186/s12889-023-16797-z

**Published:** 2023-10-04

**Authors:** Sylke V. Schnepf, Michela Boldrini, Zsuzsa Blaskó

**Affiliations:** 1 European Commission, Joint Research Centre, Ispra, Italy; 2 Bocconi University, Milan, Italy; 3 European Commission, Joint Research Centre and ICF, Brussels, Belgium

**Keywords:** School loneliness, Bullying, Teacher support, Education policy, Well-being, Europe

## Abstract

**Background:**

Loneliness has been recognized as a public health issue and has moved into a number of European countries’ policy agendas. Literature examining loneliness in young people (and especially in adolescents) is scarce, but it does show that at this age feelings of loneliness have been increasing in recent decades and are detrimental for both adolescents’ current and future well-being. In order to explain loneliness, current literature focuses generally on individual, rather than on broader, environmental characteristics. This study examines school associates of loneliness and compares their importance to those at the individual level because schools are the most important places in which adolescents are socially embedded. In addition, policy interventions on loneliness might be more feasible at the school than the individual level.

**Methods:**

This study uses a single-item measure of adolescents’ loneliness feelings in schools and exploits rich data from the Programme for International Student Assessment (PISA 2018) on 23 European countries covering 118,698 students (50.2% female) in 4,819 schools. This study applies multi-level models to investigate school level factors jointly with those at the individual level.

**Results:**

Differences between European schools can explain a 20% variation in feelings of loneliness, thereby indicating the importance of the school environment. Furthermore, adolescents’ bullying experiences and a bullying climate in school more than doubles incidences of loneliness. In addition, a cooperative climate as well as teacher support can considerably decrease school loneliness. Cross-level interactions do exist: being from a lower socioeconomic background for instance, while not important generally, increases loneliness feelings if most of the school peers are from a better socioeconomic background. School factors appear to be more important for explaining young people’s loneliness incidence than individual characteristics.

**Conclusion:**

This is the first study to compare school level and individual level factors relating to youth loneliness in schools throughout Europe. Results emphasizing the importance of school environment for explaining adolescents’ loneliness suggest that school level initiatives may be most appropriate in tackling loneliness when compared to wider and less contextualized national policies that focus on adolescents outside of school.

**Supplementary Information:**

The online version contains supplementary material available at 10.1186/s12889-023-16797-z.

## Background

Loneliness, described in the journal “The Economist” as “the epidemic of the 21st century” in 2018, is linked not only to higher risks of mental health problems, chronic illnesses, and mortality (e.g. [1]), but also to detrimental consequences on both social cohesion and trust [2]. The political arena has recognized loneliness as a public health problem that needs intervention at the societal level by creating the post of a UK (in 2018) and Japanese (2021) Minister for combatting loneliness.

Loneliness has attracted increased attention across various disciplines of research, with the focus of research shifting from the elderly population to younger age groups (e.g., [3]), including adolescents. The literature calls for a better understanding of how loneliness develops during this sensitive period of life, when loneliness appears to increase after having been at moderate levels during childhood [4].

Much like it is for all age-groups, lonely adolescents have poorer well-being and mental health [5]. Loneliness leads to depression in both the short- [6] and long-terms [7], as well as to anxiety, sleep problems, low self-esteem [8], and aggression [9]. Adolescents’ loneliness is linked to higher unemployment later in their lives [7]. Given loneliness’s dire social and health consequences, it is worrying that 15-year-olds’ feelings of loneliness in school increased in 36 out of 37 countries between 2012 and 2018 [10]. The pandemic is also likely to have given rise to further feelings of loneliness [2].

Decreasing adolescents’ loneliness is vital for improving adolescents’ well-being and mental health as well as preventing loneliness during adulthood, since adolescents’ loneliness is closely linked to their feelings of loneliness once they have grown up [11]. Which policy interventions can help to combat adolescents’ loneliness? Research on adolescents’ loneliness is still scarce. In addition, existing research has paid more attention to distinguishing between demography-, health-related, and social-environmental factors of loneliness [12], but has almost completely neglected the broader environment in which adolescents are situated.

Regarding key demography-related associates, findings on gender (see e.g., [4]; [11]) are inconsistent, probably due to how loneliness is measured. Specifically, girls appear to be more likely to describe themselves as being lonely, while the occurrence of loneliness appears to be higher among boys when measured indirectly [7]. A lower socioeconomic background is linked to higher levels of loneliness when background is measured by income [9] or by parental education [7]. One’s immigration background, and especially being a first-generation migrant, appears to be a key predictor of loneliness in adolescence in Denmark (e.g., [13]), while ethnic minority adolescents in Manchester tend to be less lonely than their local peers [14]. Regarding health-related associates, research findings suggest that shyness as well as low self-esteem are strong predictors of loneliness among adolescents [15].

The current loneliness research, with its focus on demography- and health-related factors, implicitly treats loneliness as a problem centred around the individual and less around the individual’s social embeddedness outside the family. Individual centred research can suggest solutions at the individual level, but it can only point at the target group of interventions by identifying certain individual, unchangeable demographic characteristics linked to loneliness; it cannot identify the mode of intervention itself [12]. Moreover, any policy interventions that aim to decrease loneliness are probably much easier to target and implement, and are more cost-effective at the broader environmental level compared to the individual level.

Consequently, it is important to investigate the socio-environmental risk factors of loneliness. For adolescents, schools provide a primary site of socialisation [16]; this is also where a large part of adolescents’ peer-relations are formed. Therefore, the school environment is likely to be the most significant socio-environmental context that can present risk factors of, as well as possible preventions against, loneliness. However, there is only very limited evidence about what schools can do to protect adolescents from feeling lonely at present.

Existing research on relevant school factors predominantly consider students’ individual experiences in school. A small number of studies focus on loneliness and perceived peer relations in schools (e.g. [17], [15]), indicating that perceived cooperative peer relations as well as a sense of social acceptance and higher friendship quality, are linked to lower loneliness incidence. Moreover, [18] discuss that peer relations in schools are much more important than an individual’s background. Higher teacher support, a positive classroom environment, and social support from peers go hand in hand with lower loneliness risk [19]. Regarding transitions between school types, the loneliness of adolescents drops when they switch to school types that engage in a greater level of mixing of students across the different classes [6].

While these studies provide important insights into the relevance of school environment in shaping loneliness, they do not consider students as being nested in schools. Consequently, they neither allow critics to identify school level factors associated with individual level loneliness, nor do they make it possible to assess the relative role played by schools in combatting adolescents’ loneliness. Interestingly, to the best of our knowledge, the only studies that apply multilevel approaches to the analysis of loneliness in adolescence [20] look at the role played by neighbourhoods and geographical areas as a key environmental factor that can predict loneliness. Neighbourhood-belonging explains a small (about 1%) but significant proportion of variation in loneliness among adolescents aged 12–15 years with some specific community characteristics being a significant predictor of loneliness at the individual level [14]. Moreover, individual characteristics, including ethnicity, gender identity, and sexual orientation show different associations with loneliness in different neighbourhoods; this suggests that different environments offer different opportunities for minority groups to avoid loneliness. Focusing on young adults aged 16 to 24, the geographic region is associated with 5–8% of the variation in loneliness [20]. These results stress the importance of environmental factors when explaining loneliness incidence.

However, similar questions, but which are related to the importance and the role of schools in explaining loneliness, have so far gone unanswered. This study is, therefore, novel in its consideration of schools as the key socioeconomic environment of adolescents and in applying a multilevel approach in order to understand the absolute and relative importance of schools in combatting loneliness in adolescence. It does so not only by taking into account that 15-year-olds are nested in schools, but also by studying a range of school characteristics as potential loneliness associates.

For example, we assume that the composition of students and their concomitant social segregation might be relevant, in terms of average educational achievement, as is the overall concertation of migrants in school. Literature examining educational outcomes has noted the importance of peer composition [15]. We can also assume that those school policies that increase the interaction between students (e.g., by offering extracurricular activities in school) can decrease loneliness levels by increasing peer bonding.

In line with the existing research [19], we also take students’ personal experiences in school into account and, thus, factors that can potentially be targets of school-level loneliness prevention. We look at adolescents’ perception of teacher support, their educational outcomes, their experience of grade repetition, and their experiences of bullying. In addition to the individual experiences in school, we further consider a range of socio-demographic and health-related correlates of loneliness at the individual level.

This multilevel approach allows us to consider three main research questions.

First, *which school factors are related to higher incidences of loneliness*? The school factors considered include both school characteristics and individual experiences at school.

Second, *do school and individual factors interact for explaining loneliness?* Our study’s multilevel nature allows us to consider the interplay between individual and environmental factors, as suggested by [12]. It could be that some schools are better or worse at combatting loneliness for a specific type of student (e.g., migrant students might feel lonelier in a school with a very low concentration of migrants than in a school with a very high concentration of migrants). The study examines these cross-level interactions thoroughly.

Third, *compared to the association between individual characteristics and loneliness, how sizable is the association of school factors with loneliness?* If the school environment is more important for explaining loneliness than individual characteristics, school policies can potentially be highly successful in combatting loneliness feelings.

Using rich data from the Programme of International Student Assessment (PISA 2018) and applying multi-level regression analyses allowing an examination of the individual and school level and their cross-level interactions, makes it possible to provide a multifaceted answer on how schools can decrease adolescents’ loneliness. The research adds further value by examining loneliness across 23 European countries. This is novel as existing literature is consistently based on single city or country studies, thereby providing results that might not be generalizable to the general European context.

## Methods

### Study participants

Data is derived from the 2018 Programme for International Student Assessment (PISA) survey, which was organised by the OECD. The cross-national survey on 15-year-olds in schools contains in-depth information covering their literacy skills (PISA’s main outcome variable), their socioeconomic status, attitudes and perceptions in school, and headmasters’ information on school characteristics. The survey follows a strict randomisation procedure and uses two stage sampling covering the school (applying probability proportional to size (PPS) sampling) and student level for each country in order to achieve nationally representative samples. Countries participating in PISA can exclude up to 5% of 15-year-olds with special educational needs from their sample (for more information on the survey methodology employed see Chap. 4 in [21]).

The study excludes the three PISA countries Spain, Austria, and Sweden because they did not collect information important for our analysis (such as academic skills, school size, and school location). Consequently, this study focuses on the remaining 23 European Union member countries (Belgium, Bulgaria, Czechia, Germany, Denmark, Estonia, Finland, France, Greece, Croatia, Hungary, Ireland, Italy, Lithuania, Luxembourg, Latvia, Malta, the Netherlands, Poland, Portugal, Romania, Slovakia, and Slovenia), which share similar values and common goals for education, as testified by various European Commission initiatives, like the European Education Area [22].

Participants were excluded if they did not respond to the dependent variable about feeling lonely (10% of the sample). This unusually high non-response might be linked to the stigma that is often associated with loneliness [23]. We, therefore, believe that we have underestimated the incidence of loneliness in schools. The PISA design allows countries to exclude special needs children who tend to be lonelier [24]. This is likely to contribute further to a downwards bias of our school loneliness estimate. In addition, a small number of participants were excluded for variables with very low non-response rates (below 2% for variables). Our final sample includes 118,698 students in 4,819 schools.

### Measurement

#### Dependent variable

The literature generally differentiates between “loneliness feelings” as a subjective measure of loneliness and “social isolation” capturing the absence of relationships [2]. This study looks at the subjective perception of loneliness: adolescents are asked about their level of agreements with the statement: “I feel lonely at school”. We identify those students as feeling lonely (coded as “1”) who answer “agree” or “strongly agree” (instead of “neither nor”, “disagree”, and “strongly disagree”, coded as 0).

In line with some other studies in the field (e.g., [6]) our questions focus on “loneliness in schools” instead of general loneliness. Schools provide an “ideal context for studying loneliness” [16] since, as discussed above, they represent the primary site of socialisation for adolescents in which their peer-environments are formed.

#### Independent variables: school level

At the school level, we differentiate between school characteristics and adolescents’ experiences in schools.

*Individuals’ experiences in school* cover grade repetition, whether the 15-year-old is not reaching basic reading skills (at or below PISA proficiency level 2) or is a top reader (students at PISA proficiency level 5 or above) and whether the student perceives a lack of teacher support. We build a continuous measure of bullying experience ranging from − 3 to + 3 aggregating students’ answers to three PISA items concerning bullying. Each item is recoded to take the values − 1, 0, or + 1 if students reveal that they have experienced bullying “never or almost never”, “a few times a year”, or “a few times per month or more”. The higher the value, the greater the individuals’ experience of bullying. The three items are: “Other students left me out of things on purpose”, “Other students made fun of me”, and “I was threatened by other students”. We define students to have had a moderate bullying experience in the event that the student experienced a non-negligible number of bullying episodes with values ranging between − 2 and 0 (46% in our sample), and a high bullying experience if their values are between 1 and 3 (8% of the sample).

We consider the following *school characteristics*: whether the school is situated in an urban area (compared to a school in a village, hamlet, or rural area), the school size (standardized z-scores based on the entire school sample), whether the school does ability grouping within the schools compared to no ability grouping, and the number of extracurricular activities offered.

Furthermore, we create additional school-level characteristics (proportion of low socioeconomic background students, of migrants and of grade repeaters, students perceiving low teacher support, share of students with low and top reading outcomes, share of students perceiving students in the school as cooperating or competing) by averaging individual level variables across each school if the school has at least 8 responding students. For bullying incidence, we take the mean of students’ bullying experiences (measured as discussed above) across schools. We compare schools that have moderate bullying incidence (those schools between the 25^th^ and 75^th^ percentile rank if ordered by bullying incidence) and high bullying incidence (schools above the 75^th^ percentile) with those schools in which students report only small or no bullying incidence on average (schools below the 25^th^ percentile). Having only a small sample of students per school (between 8 and 42 students) leads to a measurement error of these estimated school variables so that the association of the school variable with loneliness will be underestimated (the so-called “attenuation bias” [25]).

Sections A1 and A2 in the supplementary material provides in-depth information about the PISA questionnaire items, the construction of variables, and their codings. Section A3 provides information on number of schools in countries.

#### Independent variables: individual level

We consider the following binary variables in line with the literature that discusses risk factors related to loneliness: gender, first and second generation migrant status, whether students speak a different language at home than that of the testing language, and students’ perceived lack of parental support.

We also measure socioeconomic status dichotomously as is often done in education literature (“0” if none of the parents hold tertiary education and “1” if otherwise). We do not use PISA’s continuous summary indices of socioeconomic status since, first, these indices are not standard and, consequently, cannot be easily compared between different loneliness studies and, second, they are standardized to a completely different set of countries (OECD countries) than the ones that we focus on in our study (European countries only).

Individuals’ mental health factors can reflect both loneliness risk factors and outcomes ( [26], [4]). The direction of the association is not clear. Consequently, any “effect” of explanatory mental health variables on loneliness must be considerably upwards biased capturing also the direction that loneliness decreases mental health. Moreover, negative emotions can also be outcomes of what is happening in the school [27]. Nevertheless, given that we compare the importance of individual with school factors when examining incidences of loneliness, we also consider adolescents’ mental health as explanatory variables. Our measures are: whether students always feel afraid (9% in the sample), sad (5%), and lack self-belief to get through hard times (8%).

#### Statistical analysis

Descriptive analyses are used to describe the association of school and individual factors with loneliness. We apply nested, multi-level models to answer our research questions, thereby recognising the clustering of adolescents within schools; this is important for the measurement of accurate standard errors. In addition, the multilevel approach allows for the examination of the association with loneliness for both levels, schools as well as individuals, and their cross-level interactions.

We first test whether between school differences can explain variation in loneliness (Model 1) with a null-model. We then estimate the importance of school characteristics alone (Model 2) and in conjunction with individuals’ experiences in schools (Model 3). Model 4 includes both, all school variables of Models 2 and 3 and individual variables to compare the importance between school and individual levels. The final model adds mental health variables as an additional set of individual variables.

We apply PISA individual level weights to the two-level modelling where we consider school-level clustering. Data for the 23 European countries are merged and country fixed effects are employed.

For eight variables having item non-response greater than 2% (six school variables (area of schools, school size, competition and cooperation in schools, ability grouping in different and within classes) and two individual variables (adolescents perceiving lack of parental support and experiencing bullying)) we impute average values calculated at the country level. Imputation is indicated with a dummy variable equal to “1” if the value was imputed (“0” otherwise). For all six school variables the imputation dummy is insignificant in our regression models, indicating a possible missing at random of imputed values. However, the individual level imputation variables are strongly significant and positive. Consequently, students who choose not to answer the items on “bullying experience” and “lack of parental support” are lonelier than the average student (for in-depth results see supplementary material section A4).

Results are provided as average marginal effects from which we can derive the percentage point change in loneliness if the explanatory variable changes by one unit. All continuous variables are scaled as proportions.

Analyses were conducted with Stata 17.

## Results

### Descriptive statistics

Table 1 provides school characteristic descriptives at the school level and school experience and individual characteristics at the individual level. 13% of 15-year-olds feel lonely in European schools. Focusing on *demography-related associates*, among the 13% of students who perceive to lack parental support 22% feel lonely compared to only 11% who receive parental support (87% in the sample). 20% of first generation migrants (3% in the sample) feel lonely, compared to just 12% of second generation migrants (7% in the sample) and 13% of natives (90% in the sample). Other group differences, such as gender and socioeconomic background, are less sizable.

**Table 1:**
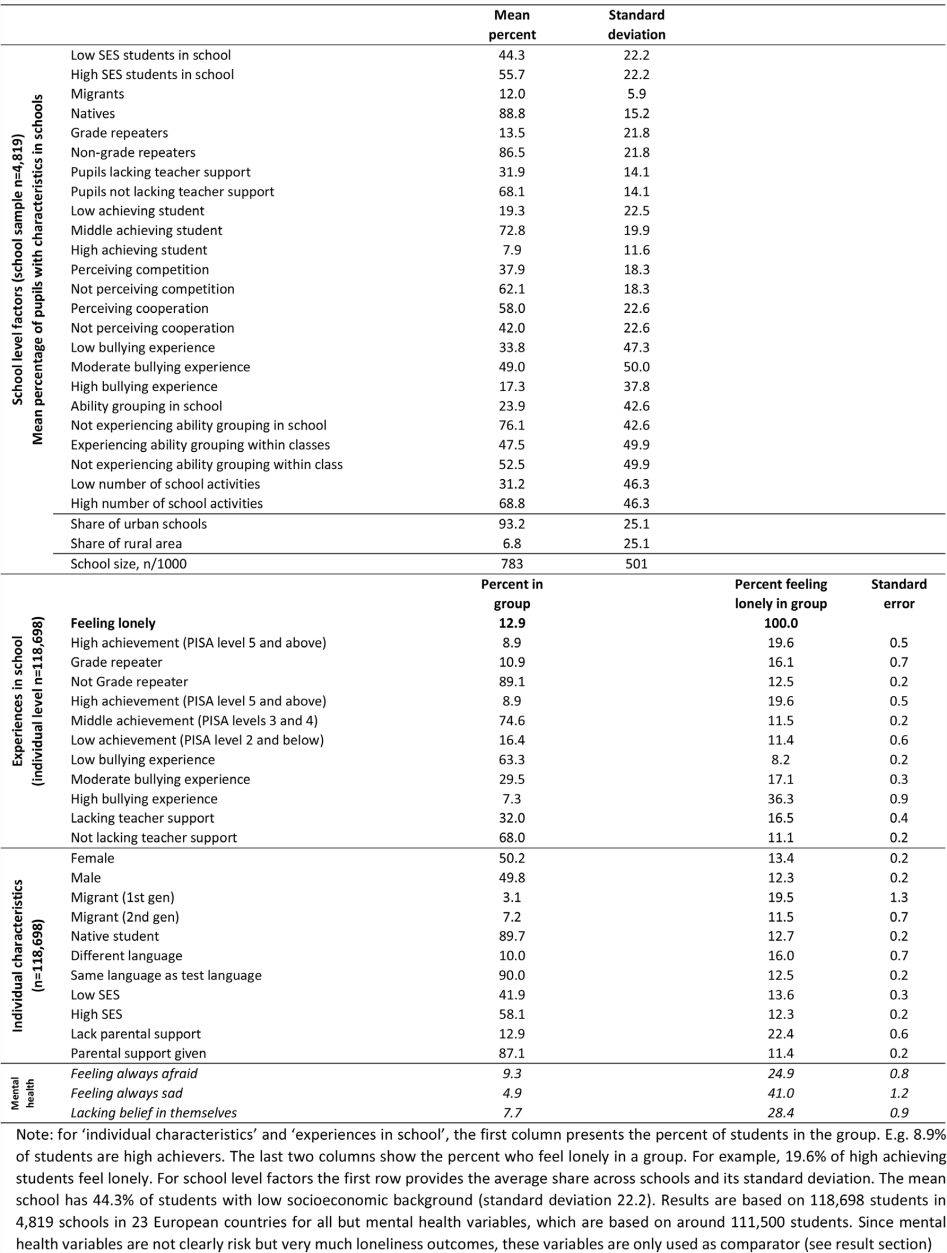
Descriptive summary statistics, 23 European countries in PISA, 2018

Focusing on *socio-environmental associates*, as many as 36% of European students who are bullied most frequently (7% in the sample) feel lonely in school, compared to 17% of students experiencing some bullying, and 8% never being or only marginally being bullied. Low achievers, grade repeaters, or students perceiving lack of teacher support feel significantly lonelier by 2 to 4 percentage points when compared to their comparison group.

**Fig. 1:**
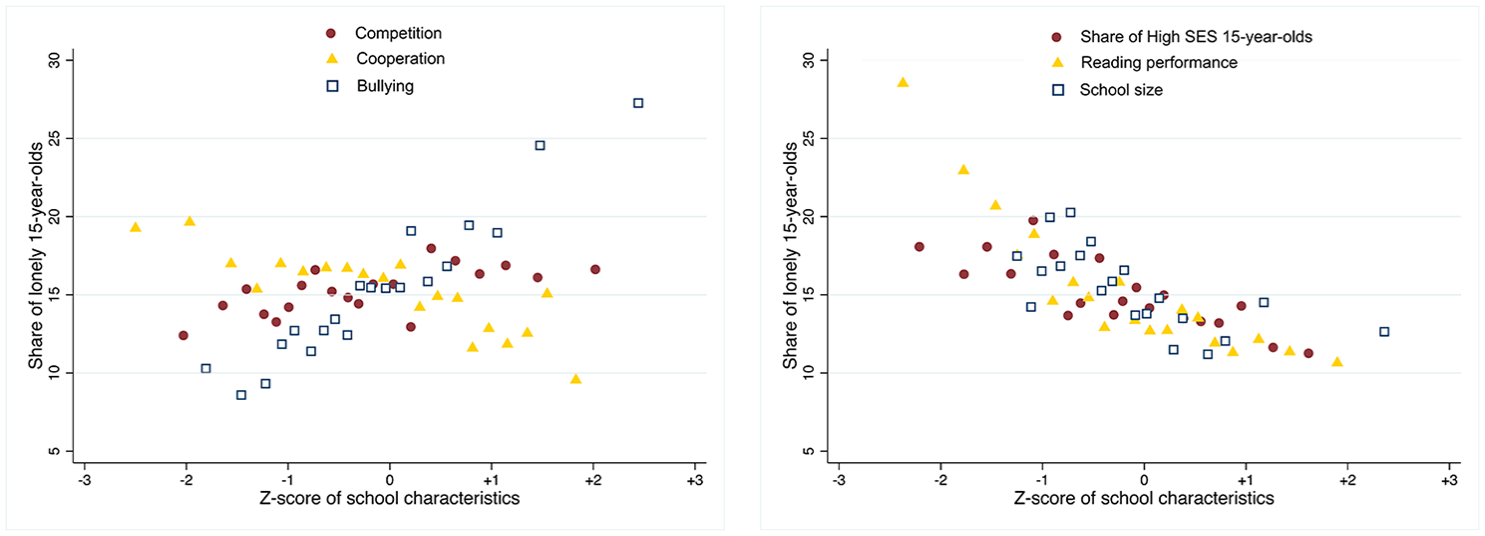
School characteristics and percent of 15-year-olds in school feeling lonely, 2018. Source: PISA 2018, authors’ calculations. European countries included are Belgium, Bulgaria, Czechia, Germany, Denmark, Estonia, Finland, France, Greece, Croatia, Hungary, Ireland, Italy, Lithuania, Luxembourg, Latvia, Malta, Netherlands, Poland, Portugal, Romania, Slovakia and Slovenia. Note: the figures provide results of binned scatterplots, which group the x-axis variable into equally sized bins (quantiles): every data point condenses information on the average value for the x- and y-axis variables within each bin. A z-score of 0 means that the school performs at the average level of the 4 819 European schools. A higher (lower) z-scores means that the respective school characteristic has also a higher (lower) than average incidence. Binned scatterplots are obtained from standardized school level data (N = 4819), weighted using PISA school weights. School level data either refer to school-specific characteristics (school size) or to simple averages (reading performance) or shares (high-SES students, *strong* perceived competition, *strong* perceived cooperation, *strong* bullying experience) of students with the underlying characteristics at the school level. The standardization is done within the 23 EU countries sample used also for the rest of the analysis

Figure 1 visualises the relationship by providing binned scatter plots to summarise those school characteristics that unconditionally appear to be most associated with loneliness. The level of bullying appears to constitute the greatest risk factor for loneliness in schools. More than a quarter of students report feeling lonely in the approximately 2.5% of schools with the highest levels of bullying (z-score of around 2 and over). This compares to ‘just’ one tenth of students in the 2.5% of schools with the lowest levels of bullying (z-score<=-2). A cooperative environment in schools is also linked to lower levels of loneliness. In contrast, schools with higher competition appear to have higher levels of loneliness. There is also a slight association of schools’ socioeconomic background composition, reading performance and school size.

### Multi-level models

#### Research question 1: which school factors are related to higher loneliness incidence?

The multi-level model results are summarised in Table 2.

**Table 2:**
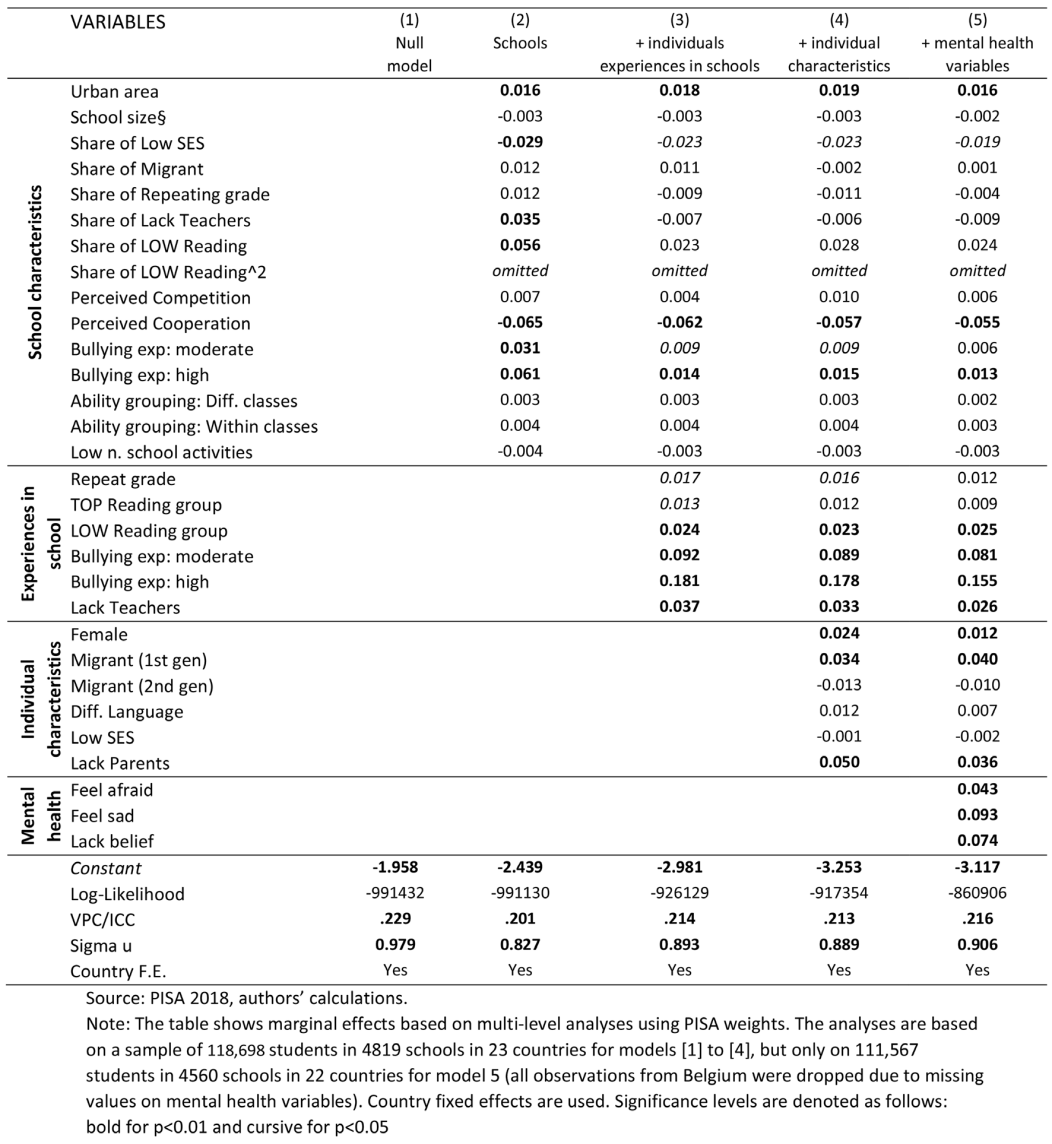
Extraction of multilevel regression results showing marginal effects

The size of the school, the number of extracurricular activities offered in the school, ability grouping (whether within or between classes), the schools’ overall intake of migrants, the share of students’ repeating a grade, and perceiving their peers to be competitive are not correlated with students’ feelings of loneliness (Model 2). In contrast, cooperation between peers seems to be what is most important. Compared to an average school, a school in which perceived cooperation increases by one standard deviation (so by 22.6 percentage points, see Table 1), loneliness in the school decreases by about 1.5 percentage points (0.065*0.226) on average. However, the degree of perceived competition seems not to influence overall incidences of loneliness.

Bullying incidence in schools is important even conditional on other school characteristics. On average, the 25% of schools with the highest bullying incidence have students about 6 percentage points lonelier than schools in the lowest quartile of bullying incidence. Furthermore, schools’ intake of low socioeconomic students matters.

Focusing on an *individual’s school experience* (Model 3) shows that being frequently bullied increases loneliness by as much as 18 percentage points and being moderately bullied by 9 percentages points compared to those with no or only limited bullying experience, keeping other school factors constant (Model 3). Conditional on school experience, the association of the share of bullied students in the school with loneliness incidence decreases significantly (comparison between Model 2 and Model 3), showing that it is the experience of being bullied, and not exposure in school, which links closer to loneliness feelings.

Students who perceived their teachers as unsupportive are, on average, 4 percentage points lonelier than students who find their teacher to be supportive. Having repeated a grade also increases loneliness by about 2 percentage points. Furthermore, being either a top or a low reading performer increases loneliness compared to performing in the middle range. However, loneliness incidence is higher for lower achievers.

Focusing on *individual characteristics*, Model 4 shows that pupils who perceive parents as non-supportive are about 5 percentage points lonelier. Being a first generation migrant increases loneliness feelings by 3 percentage points and being a girl by 2 percentage points. There is no association with loneliness for second generation immigrants, students with a lower socioeconomic status, and students who speak a different language at home than that of the test country conditional on all the other individual and school factors.

#### Research question 2: do school and individual factors interact for explaining loneliness?

School and individual level factors might be interacting. Regression results, using cross-level interactions, are provided in the supplementary material A4.

**Fig. 2:**
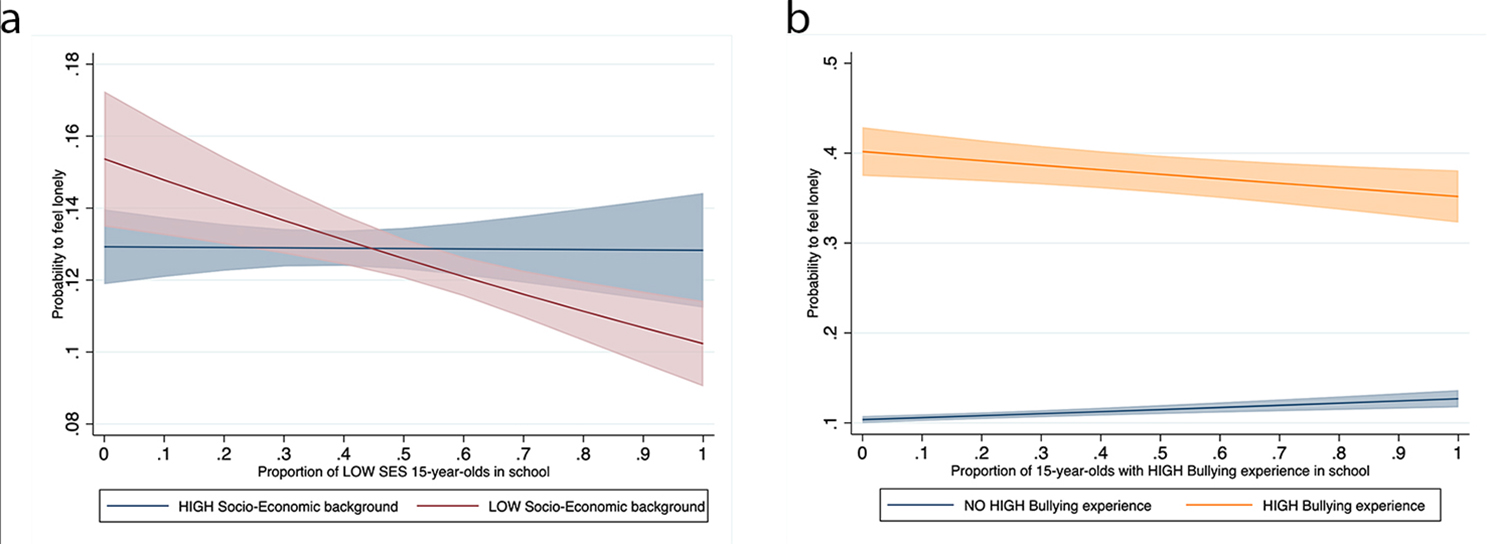
Cross-level interaction **a**) Loneliness in school of students with lower socioeconomic background by schools’ overall share of lower socioeconomic background students. **b**) Loneliness in school of students with severe bullying experience by schools’ overall share of severely bullied students. Note: the graphs plot marginal effects of being in a school with a certain proportion of lower socioeconomic background students (a) or of bullying incidence (b) for 15-year-olds by own low or high socioeconomic background (a) or high or no bullying experience(b) on the probability to feel lonely

Figure 2 visualises significant cross-level interactions found. Students from lower socioeconomic background feel lonelier than other students in schools where the share of low socioeconomic backgrounds students is low, while they feel less lonely in schools with higher intake of lower socioeconomic background students. We do not find a similar pattern for adolescents from a higher socioeconomic background though. In addition, results show a significant interaction between individual level bullying experiences and a school’s proportion of 15-year-olds with experiences of bullying. Those who are bullied feel less lonely in a school with higher, than with a lower, incidence of bullying. Seeing that many peers are bullied might decrease the value attached to experiencing yourself bullying. Adolescents with no bullying experience in contrast feel lonelier in schools with a higher incidence of bullying.

Furthermore, we investigated whether students who are top performers feel lonelier in schools with many low performers, whether low performers feel lonelier in schools with more top performers, and whether migrants feel lonelier in schools having a comparatively smaller share of migrants. We did, in fact, find the assumed direction for all three cross-level interactions. However, the significance level for these three interactions was only 10% and this appears to be a very low benchmark for discussing a clear association, given our huge sample size of almost 120,000 students in 5,000 schools.

#### Research question 3: compared to the association between individual characteristics and loneliness, how sizable is the association of school factors with loneliness?

The Variance Partition Coefficient (VPC) of the Null-model (Model 1 in Table 2) indicates that 23% of the variation in student loneliness is due to variation among European schools (country fixed effects are taken into account for this estimate). Running Null-models at the country level shows huge variation with highest VPCs in Germany and the Netherlands (around 60%) and lowest in Bulgaria, Croatia, Ireland, Malta, and Slovenia (around 10%). Clearly, schools matter considerably beyond individual factors when explaining the subjective feeling of loneliness.

As discussed above, individuals’ mental health factors can include both loneliness risk factors and outcomes [26]. Nevertheless, given that we compare the importance of individual and school factors for examining loneliness incidence, it could be argued that we should consider even upwards biased mental health associations. This is done in Model 5 (Table 2). We consider three variables: whether students always feel afraid (9% in the sample, see Table 1), sad (5%), and lack believing in themselves for getting through hard times (8%). Model 5 in Table 2 shows that all measures are closely associated with loneliness, conditional on all other individual and school factors.

Nevertheless, bullying is across both levels, school and individual, the key determinant of feeling lonely, even once mental health factors are taken into account. Given an average loneliness of 12.9%, an increase in loneliness by 16 (Model 5 assuming mental health factors only cause loneliness and are not consequence thereof) or 18 percentage points (Model 4 unconditional on mental health factors) for those experiencing frequent bullying is exceptionally high.

In second place come mental health issues, assuming that they are only risks and not consequences of loneliness (which is not true). Adolescents who always feel sad have on average a 9 and adolescents lacking belief in themselves have a 7 percentage points higher probability of feeling lonely.

The highest, third association concerns cooperation in school: adolescents in a school with comparatively lowest perceived cooperation among students fare about 6 percentage points worse in loneliness incidence than adolescents in schools with highest levels of cooperation.

## Discussion

Existing research has focused on demography and health-related associates of adolescents’ loneliness, thereby neglecting social-environmental associates [12]. To the best of the authors’ knowledge, this is the first study to explore the association of numerous school characteristics and experiences with loneliness across European adolescents in schools, thereby comparing the importance of individual and social-environmental associates.

### Prevalence of loneliness in schools

Our study showed that 13% of 15-year-olds felt lonely in schools throughout Europe in 2018. Nevertheless, we assume that our estimate is downwards biased given the PISA’s survey design (excluding children with learning disabilities who tend to feel lonelier [28] and an unusually high 10% non-response on the loneliness question probably due to stigma). Previous studies which also used single-item loneliness questions about overall (in contrast to school) loneliness among European young adults indicated a loneliness incidence of 9% in 2016 ( [2], 18 to 25-year-olds) and 20% in 2022 ( [29], 16 to 25-year-olds).

### The school as environmental associate of adolescents’ loneliness

The school environment is likely to be the most significant socio-environmental context for adolescents. Multi-level results (the Null model) showed that as much as 23% of the variation in adolescents’ loneliness is due to variation among European schools. Previous studies found that geographic region explains about 5–8% [20] and neighbourhood about 1% [14] of young people’s overall (in contrast to school) loneliness. Schools’ higher importance can be explained by schools’ more confined composition within which networking is clearly defined (compared to geographic area or neighbourhood) and the focus on the more distinct notion of school loneliness. Consequently, schools matter and can make a difference to how adolescents feel.

After dividing school associates of loneliness into school characteristics and students’ experiences in schools, results showed that it is the latter that matter most: students who are exposed to frequent bullying in school are the loneliest. Even conditional on a huge number of other school and individual characteristics, those 7% of European 15-year-olds who are regularly bullied have an over 2.5 times higher probability of being lonely than the general population. Once students experience some level of bullying (30% of the population) loneliness increases by 9 percentage points. The school level occurrence of bullying remained significant and sizable conditionally on students’ individual level bullying experiences: a school with high bullying incidence has, on average, adolescents being 1 percentage point lonelier compared to other schools. This clearly shows that bullying is harmful for the entire school community and not just its direct victims.

Furthermore, and in line with findings by [15], those schools perceived by students as having a cooperative climate have, on average, considerably lower loneliness incidence than schools in which peers appear less cooperative. We also confirmed results by [19] that showed that teacher support matters: European students who perceived their teachers as not being supportive are 3 percentage points lonelier on average (conditionally on all school and individual factors) compared to students who felt supported. In addition, students who experienced grade repetition, schools with a high share of lower performing students, and urban schools have slightly elevated levels of loneliness. Loneliness incidence is also higher for low achievers. This result confirms the idea behind the ‘whole school approach’ that recognises the close link between academic learning and emotional health [30]. It is notable that the “effects” of school characteristics and experiences in school with loneliness, as reported above, remained stable even where we included controls for individual mental health (even though the latter are, at least partially, outcomes of loneliness and are subject to endogeneity).

Nevertheless, and against expectations, some school characteristics were not associated with incidences of loneliness, like the schools’ size and number of extracurricular activities offered. We also could not confirm that school type matters for incidences of loneliness [6], given that ability grouping practices in schools were not found to be associates of loneliness.

### Individual level association

The predominant part of the literature on adolescents’ loneliness focuses on demography-related associates. The results supported the finding [7] that females feel lonelier than males (by 1 percentage point). In line with the literature [13] migration status matters, but only first, and not second generation, migrants are lonelier than natives (by about 4 percentage points). However, the most important conditional associate of loneliness was not adolescents’ demographic background, but rather their relationship with their parents: adolescents perceiving their parents as supportive are 4 percentage points less lonely than their peers with less supportive parents.

### Cross-level interactions

Cross-level interactions measure whether school and individual level associates with loneliness are interlinked. Results showed that disadvantaged adolescents feel lonelier in schools in which most peers are advantaged, but who are less lonely provided that their peers come from a socioeconomic background similar to theirs. Similarly, but only at a 10% significance level, we found that migrants feel lonelier in schools that have a comparatively smaller share of migrants. This interaction between migration status and school composition could perhaps explain the contradictory results in the literature about how migration and ethnicity are linked to loneliness. Moreover, the cross-level interaction shows that students who differ from the majority of students attending the school might need additional support.

Furthermore, students experiencing bullying feel lonelier in schools with low (compared to those with high) incidences of bullying, whereas those with no bullying experience feel lonelier in schools with high (compared to low) shares of students being bullied. Consequently, the severity of the bullying experience depends on a school’s individual contextual factors.

### School and individual associates compared

 [18] showed that peer relationships are more important than individuals’ background for explaining incidences of adolescents’ loneliness by using data for 8 schools in Turkey. This study examined the importance of school and individual level associates with loneliness in the European context.

School characteristics and experiences in school are much more important for explaining school loneliness than individual demographic factors. Not only can schools explain one quarter of the variation in students’ feelings of loneliness, but bullying experience in the school, rate of bullying in schools, the absence of a cooperative environment, and teacher support are highly associated with incidences of loneliness. The most important family background associate with loneliness is parental support, in contrast to demographic associates like gender and migration background.

Given this result, existing literature focusing predominantly on individual associates of loneliness might neglect the most important drivers of adolescents’ loneliness (as has been previously argued by [12], [14]).

### Implications for policy

As discussed by [12], the individual centred research on adolescents’ loneliness that exists at present could only identify certain individual (often unchangeable) characteristics linked to loneliness and, thus, only point at the target group of policy interventions, not to its mode. For policy initiatives, it is necessary to understand the level at which the action lies, and hence the level at which interventions are most likely to succeed.

Results showed that schools are important players for decreasing loneliness, thereby making them a good target for education policies. Education policies and interventions that aim to decrease loneliness should first tackle incidences of bullying in schools, should foster a more cooperative climate between students’ peers, and should promote teacher support for students in the school. Both big schools and small schools can undertake this task, but lower achievement in the school and grade repetition practices are linked to higher incidences of loneliness.

### Limitation

The study has a number of limitations. First, and most importantly, it cannot identify either causality or the direction between loneliness and its associated factors. Second, almost 10% of students did not answer the loneliness question; this could be due to a stigma associated with loneliness and the result is that we underestimate loneliness incidence in Europe. Third, about 23% of the variation in adolescents’ loneliness can be explained by school variation. However, once we take schools’ characteristics into account this percentage decreases only marginally. This indicates that even though we have rich data on European schools, we lack information about school factors that are important for explaining loneliness. Fourth, coefficients of those school characteristics estimated by averaging across the PISA sample (e.g. share of adolescents’ bullied in school) suffer of downward attenuation bias due to measurement error in the variable. Consequently, the study underestimates the importance of schools in our modelling. Fifth, generalisations of the results to European 15-year-olds would be flawed because we focus on 23, instead of 27, European countries as members in the European Union. Therefore, the omitted countries might differ in their association of loneliness with its attributes. Sixth, the study focuses only on 15-year-olds and not on a wider age group of adolescents. Seventh, we focus on a single item measure of school loneliness which we summarise in a dichotomous variable. A recent study compares the De Jong-Gierveld (DJG) 6-item scale, University of California Los Angeles (UCLA) 3-item scale and a single item question on loneliness and yields very similar results [31]. Last, variables measuring internet use among 15-year-olds were missing for almost 13% of our PISA sample. Four European countries (Germany, the Netherlands, Portugal, and Romania) did not collect any data on the topic at all. We considered these countries to be important to include in our European sample of adolescent students. Consequently, the study does not investigate internet use, even though it is associated with incidences of loneliness among adolescents [32].

### Implications for future research

Given the importance of schools when explaining incidences of loneliness among adolescents, future research would benefit from investigating environmental associates with loneliness further. In addition, results indicated that the link between an individual’s set of characteristics and loneliness is dependent upon the specific school and environmental context. Consequently, future research could provide further interesting results by investigating these cross-level interactions.

While our results indicated that the school level is a promising level at which to analyse loneliness interventions, the inclusion of a wide range of school variables could not substantially decrease the very high VPC. This could be due to a lack of data on schools’ curricula regarding social emotional learning and information about other school activities that aim to improve students’ mental health and resilience (such as the availability of student support).

## Conclusion

This is the first study to compare school level and individual level factors related to youth loneliness in schools throughout Europe. Results emphasise the importance of school differences when explaining adolescents’ loneliness and suggest that school level initiatives may be most appropriate in tackling loneliness compared to wider (and less well contextualised) national policies that focus on adolescents outside of school.

### Electronic supplementary material

Below is the link to the electronic supplementary material.

Supplementary Material 1

## Data Availability

The secondary datasets analysed for the current study are available from. https://www.oecd.org/pisa/data/2018database/. The Stata 17 do-files used for producing the results are available on request from the authors.

## Declarations

### Disclaimer

The views expressed are purely those of the authors and may not in any circumstance be regarded as stating an official position of the European Commission.
