# Supplementary material for: Adolescents’ loneliness in European schools: a multilevel exploration of school environment and individual factors
Source: BMC Public Health. 2023 Oct 4;23:1917. doi: 10.1186/s12889-023-16797-z (PMC10548635; doi:10.1186/s12889-023-16797-z)
Supplement: Supplementary file 1 — Supplementary Material 1 [file 12889_2023_16797_MOESM1_ESM.docx]

**Adolescents’ loneliness in European schools:**

**a multilevel exploration of school environment and individual factors**

*Sylke V. Schnepf, Michela Boldrini and Zsuzsa Blaskó*

September 2023

**Supplementary material**

[(A1). Description of the missing values handling strategy 2](#_Toc142995382)

[Table A1a: Results of the missing values handling strategy by country 4](#_Toc142995383)

[Table A1b: Independent variables with missing and imputed values 5](#_Toc142995384)

[(A2). Variable description 6](#_Toc142995385)

[(A3). Descriptive statistics: School-level data 10](#_Toc142995386)

[Table A3: Number of schools by country (N=4819) 10](#_Toc142995387)

[(A4). Additional results from multilevel regressions 11](#_Toc142995388)

[Table A4.1: Extraction of multilevel regression results showing estimated coefficients 11](#_Toc142995389)

[Table A4.2.a: Extraction of multilevel regression results with interactions (est. coeff) 13](#_Toc142995390)

[Table A4.2.a: Extraction of multilevel regression results with interactions (est. coeff) 15](#_Toc142995391)

# (A1). Description of the missing values handling strategy

We followed the following strategy to handle the presence of missing values in our data. All the two dropping/imputation operations listed below are carried out in this subsequent order.

1. Missing data on the dependent variable and on the main independent variable of interest (reading performance): observations are dropped.

- (1a) Dependent variable “Loneliness”: we drop all observations for which data on loneliness are not available (after this stage, the sample counts N = 171334 observations).
- (1b) Independent variable of special interest “Reading performance”: this information is missing for Spain, we drop all observations from Spain (after this stage, the sample counts N = 138592 observations).

2. Missing data on other independent variables: if the missing values rate is below 2% observations are dropped; if the missing values rate is above 2%, missing values are imputed with country average (for continuous variables) or modal values (for categorical variables).

After this stage, the sample counts N = 132730 observations from 25 countries.

*List of vars with missing values rates*

*Missing value rates < 2%*

- Diff. Language: 211 (0.15%) missing values, observations dropped
- Repeat: 293 (0.21%) missing values, observations dropped
- Lack Teachers: 1391 (1.00%) missing values, observations dropped
- Migrant: 2519 (1.95%) missing values, observations dropped
- Low SES: 1555 (1.32%) missing values, observations dropped
- Share of Migrant: 5 (0.00%) missing values, observations dropped

*Missing value rates > 2% (Imputed)*

*- Continuous vars:*

- School size: 14105 (10.18%) missing values, set to country average value

*- Categorical vars:*

- Lack Parents: 10591 (7.64%) missing values set to country modal value
- Urban area: 9457 (6.82%) missing values set to country modal value
- Ability grouping: Diff. classes: 10145 (7.32%) missing values set to country modal value
- Ability grouping: Within classes: 7499 (5.41%) missing values set to country modal value
- Perceived Competition: 12705 (9.17%) missing values set to country modal value
- Perceived Cooperation: 17555 (12.67%) missing values set to country modal value
- Bullying Experience - item 1: 15212 (10.98%) missing values set to country modal value
- Bullying Experience - item 2: 15647 (11.29%) missing values set to country modal value
- Bullying Experience - item 3: 15852 (11.44%) missing values set to country modal value
- Feel afraid: 8866 (6.40%) missing values set to country modal value^[[1]](#footnote-1)^
- Feel sad: 8778 (6.33%) missing values set to country modal value^[[2]](#footnote-2)^
- Lack belief: 4524 (3.26%) missing values set to country modal value

3. Missing data or low-quality data on school-level independent variables: (1) We exclude observations for which all school-level variables of interest^[[3]](#footnote-3)^ are not available; as a result, the total count of observations shrinks to 121961, from 23 countries. (2) We exclude observations on students enrolled in schools for which less than 8 observations are available in the sample (the cut-off chosen for the students-per-school count is set equal to the mean value over the pooled sample minus one standard deviation). As a result, the final count of observations in the sample is equal to 118698 from 23 countries

## Table A1a: Results of the missing values handling strategy by country

| COUNTRY | SAMPLE SIZE – at the different steps of the missing values’ handling strategy | | | | |
| --- | --- | --- | --- | --- | --- |
|  | Step-0.  PISA2018-EU | Step-1a.  Loneliness | Step-1b.  Reading | Step-2.  MissingRate < 2% | Step-3.  School data  [**Final sample**] |
| AUT | 6802 | 6273 | 6273 | 6051 | 0 |
| BEL | 8475 | 7560 | 7560 | 7163* | 7131 |
| BGR | 5294 | 4237 | 4237 | 4010 | 3919 |
| CZE | 7019 | 6410 | 6410 | 6206 | 6020 |
| DEU | 5451 | 3888 | 3888 | 3633 | 3512 |
| DNK | 7657 | 6429 | 6429 | 6214 | 6113 |
| ESP | 35943 | 32742 | 0 | 0 | 0 |
| EST | 5316 | 5078 | 5078 | 4995 | 4798 |
| FIN | 5649 | 5235 | 5235 | 5090 | 5068 |
| FRA | 6308 | 5611 | 5611 | 5398 | 5289 |
| GRC | 6403 | 6020 | 6020 | 5880 | 5750 |
| HRV | 6609 | 6236 | 6236 | 6089 | 6076 |
| HUN | 5132 | 4858 | 4858 | 4791 | 4548 |
| IRL | 5577 | 5332 | 5332 | 5115 | 5115 |
| ITA | 11785 | 10548 | 10548 | 10238 | 9976 |
| LTU | 6885 | 6329 | 6329 | 6097 | 5707 |
| LUX | 5230 | 4786 | 4786 | 4421 | 4406 |
| LVA | 5303 | 4945 | 4945 | 4842 | 4547 |
| MLT | 3363 | 3061 | 3061 | 2958 | 2950 |
| NLD | 4765 | 3737 | 3737 | 3626 | 3613 |
| POL | 5625 | 5436 | 5436 | 5286 | 5227 |
| PRT | 5932 | 5437 | 5437 | 5279 | 5153 |
| ROU | 5075 | 4892 | 4892 | 3548 | 3437 |
| SVK | 5965 | 5395 | 5395 | 5275 | 4751 |
| SVN | 6401 | 5899 | 5899 | 5807 | 5592 |
| SWE | 5504 | 4960 | 4960 | 4718 | 0 |
| TOTAL | 189468 | 171334 | 138592 | 132730 | 1. 121961 2. 118698 |
| N. countries | 26 | 26 | 25 | 25 | 23 |

*Notes.* BREAKDOWN of the results of the missing values handling strategy, by country.

*“*Step-0: PISA2018 (EU)” Sample size of all EU countries in the PISA2018.

*“*Step-1a: Loneliness info” = Step-0 + dropping obs. missing info on 15yos’ loneliness status

*“*Step-1b: Loneliness info” = Step-1a + dropping obs. missing info on reading performance (ESP is excluded)

“Step-2: MissingRate < 2%” = Step-1b + dropping obs. missing info on independent variables, whose overall missing values rate is below 2% | ^#^ Belgium lacks information on 15yos self-reporting to feel ‘Sad’ or ‘Afraid’; these obs. are not dropped since these variables are used only in secondary analyses.

“Step-3: School data” = Step-2 + dropping obs. (i) missing info on school-level variables of interest and (ii) from 15yos enrolled in schools counting less than 8 students in the sample (AUS and SWE are excluded)

## Table A1b: Independent variables with missing and imputed values

| VARIABLES | Share of obs with missing (hence imputed) values in the final sample | |
| --- | --- | --- |
|  | Individual level data | School level data |
| *School level factors*  Urban area  School size, n/1000  Perceived Competition  Perceived Cooperation  Ability grouping: Diff. Classes  Ability grouping: Within Classes | 3% (3.41)  6% (5.75)  9% (8.75)  12% (12.25)  7% (7.20)  6% (5.52) | 4% (4.19)  7% (6.76)  0%  0%  8% (8.03)  6% (6.18) |
| *Individual experiences*  Lack Parents  Bullying experience (Moderate or High)    Feel afraid (Always)  Feel sad (Always)  Lack of Belief in myself | 7% (7.38)  12% (11.85)    6% (6.01) - *Not imputed*  6% (6.01) - *Not imputed*  3% (3.24) |  |

*Notes.* Shares computed on school-level data are only reported for school-level variables.

None of the “School experiences” variables have missing (hence imputed) values.

*Perceived cooperation and competition are individual-varying variables, hence within the same school it may be some 15yos’ answers are imputed while others not. For any school, for which at least one student answer was imputed we create a school dummy indicating imputation. Overall, out of 4819 schools, for 1572 and 1200 schools, respectively, values for perceived competition and cooperation are never imputed hence school-level aggregates do not count any imputed observation. At the other opposite, only for 1 and 2 schools, respectively, all the values used to compute school-level aggregates are imputed. The rest of school-level aggregates are obtained relying on varying percentages of imputed values at the individual-level. Since the imputation took place at the individual level, no school level imputation is provided in the table.

# (A2). Variable description

| **Dependent variable** | | | | | | |
| --- | --- | --- | --- | --- | --- | --- |
| Loneliness | | Feeling lonely | | Binary = 1 if students either 'Agree' or 'Strongly Agree' to the statement "Thinking about your school: I feel lonely at school". | | |
|  | | In individual-level regressions:  Y: Binary var = 1 if student self-reports to feel lonely | | | | |
| **Independent variables: *School factors*** | | | | | | |
| Urban area | | | | | | Binary = 1 if the school is located in an urban area.  *Baseline case:*  *The school is in a village, hamlet or rural area (counting fewer than 3000 people)* |
| School size*^§^* | | | | | | Sum of the total school enrolment for boys and girls as of February 1, 2018 (number of students)  *§: Standardized at school level within the 23EU sample* |
| Share of Low SES students | | | | | | School-level share of students with a Low SES |
| Share of Migrant | | | | | | School-level share of migrant students (either 1st or 2nd generation migrants) |
| Share of Repeating grade | | | | | | School-level share of students repeating grades |
| Share of Lacking Teachers | | | | | | School-level chare of students feeling lack of teachers support in school |
| Share of LOW achieving students | | | | | | School-level share of students belonging to the worst-performing group for reading achievement, PISA level <2 |
| Share of MEDIUM achieving students | | | | | | School-level share of students belonging to the mid-performing group for reading achievement, PISA level within [2;4] |
| Share of HIGH achieving students | | | | | | School-level share of students belonging to the best-performing group for reading achievement, PISA level >=5 |
| Perceived Competition | | | Share of students perceiving ‘strong’ competition feelings at school | | | School-level share of students who report the following statement is either ‘Very True’ or ‘Extremely true’: “*Think about your school, how true: It seems that students are competing with each other”* |
| Perceived Cooperation | | | Share of students perceiving ‘strong’ cooperation feelings at school | | | School-level share of students who report the following statement is either ‘Very True’ or ‘Extremely true’: “*Think about your school, how true: It seems that students are cooperating with each other”* |
| Bullying experience at the school level | | | School-level average of students’ experience of bullying in school:  School-level average of the continuous measure of bullying experience built at the individual level, ranging [-3;3]. | | | |
|  | | | LOW incidence of Bullying Experiences at the school level | | | Binary = 1 if the school falls within the Bottom-25pc of the distribution of the school-level bullying. |
|  | | | MODERATE incidence of Bullying Experiences at the school level | | | Binary = 1 if the school falls within the Middle-50pc of the distribution of the school-level bullying. |
|  | | | HIGH incidence of Bullying Experiences at the school level | | | Binary = 1 if the school falls within the Top-25pc of the distribution of the school-level bullying.  *Baseline: Low s*chool-average bullying  *(Binary = 1 if the school belongs to the Bottom-25pc of the distribution)* |
| Presence of ability grouping schemes within school | | | Ability grouping: Different classes | | | Binary = 1 if students are grouped by ability into different classes for at least some subjects |
|  |  |  | Ability grouping: Within classes | | | Binary = 1 if students are grouped by ability within classes for at least some subjects |
| Number of extra-curricular activities at school | | | Low n. school activities | | | Binary = 1 if the number of extracurricular activities offered by the school is low, such that the school belongs to the bottom 25% in the school-level distribution for number of activities offered.  *Baseline: The school offers a decent/high number of activities* |
| **Independent variables: School Experience factors** | | | | | | |
| Grade repetition | Repeat | | | | Binary = 1 if the student has ever repeated a grade | |
| Reading performance | LOW Achieving group | | | | Binary = 1 if students belong to the worst performing group. “Worst” performers belong to PISA proficiency levels below 2, where proficiency levels are based on students’ reading score and range [0;6].  Baseline: PISA proficiency levels 2-4 | |
|  | HIGH Achieving group | | | | Binary = 1 if students belong to the best performing group. “Top” performers belong to PISA proficiency levels 5 or above, where proficiency levels are based on students’ reading score and range [0;6].  Baseline: PISA proficiency levels 2-4 | |
| Bullying  Experience | Bullying Experience:  We build (in-house) a continuous measure of bullying experience, ranging [-3;3], aggregating by summation students’ answers to the three questionnaire items concerning bullying. Each item is recoded so to take values [-1;0;+1] if students reveal to have experienced bullying – respectively - never or almost never / a few times a year / a few times per month or more, so that the higher the value the greater individual exposure/experience of bullying.  The three questionnaire items used are:  - "Other students left me out of things on purpose";  - "Other students made fun of me";  - "I was threatened by other students". | | | | | |
|  | Bullying experience: Moderate | | | | Binary = 1 if students’ compact measure of bullying signals the student experienced a non-negligible number of bullying episodes over the year, hence the students’ record for the bullying experience falls within: [-2;0] | |
|  | Bullying experience: High | | | | Binary = 1 if students’ compact measure of bullying signals the student experienced a notable number of bullying episodes over the year, hence the students’ record for the bullying experience falls within: [1;3]  Baseline case:  Low bullying experience: [-3] | |
| Relationship with: Teachers | Lack Teachers | | | | Binary = 1 if students either 'Disagree' or 'Strongly Disagree' to the statement “Thinking of past two <test language lessons>: I felt that my teacher understood me.” | |

| **Independent variables: Individual factors** | | |
| --- | --- | --- |
| Gender | Female | Binary = 1 if gender is female |
| Immigrant status | Migrant | Binary var = 1 if students are either 1st or 2nd generation migrants (hence both parents born abroad) |
|  | Migrant (1st gen) | Binary var = 1 if students 1st generation migrants |
|  | Migrant (2nd gen) | Binary var = 1 if students 2nd generation migrants |
| Language spoken | Diff. Language | Binary var = 1 if students’ language spoken at home is not the testing language |
| Socio-Economic Status (SES) | Low SES (Socio Economic Status) | Binary var = 1 if neither of students’ parents has Higher Education |
| Relationship with: Parents | Lack Parents | Binary = 1 if students either 'Disagree' or 'Strongly Disagree' to the statement “My parents support me when I am facing difficulties.” |

| **(Additional) independent variables: MENTAL HEALTH** | | |
| --- | --- | --- |
| Feeling Afraid | Binary = 1 if students answer 'Always’ to the question: “Thinking about yourself and how you normally feel: how often do you feel AFRAID?” | Missing values, not imputed:  6% (6.01) – Belgium |
| Feeling Sad | Binary = 1 if students answer 'Always’ to the question: “Thinking about yourself and how you normally feel: how often do you feel SAD?” | Missing values, not imputed: 6% (6.01) – Belgium |
| Lack of Belief | Binary = 1 if students ‘Strongly Disagree’ with the statement: “How much do you agree with the following statement: My belief in myself gets me through hard times” | Missing values (imputed to country modal values):  3% (3.24) |

| **Imputation dummy variables** | |
| --- | --- |
| Imputed:  School size | Binary = 1 if school-level variable measuring school size is missing and imputed (to country average value) |
| Imputed:  Urban | Binary = 1 if school-level variable identifying school’s urban vs. rural location is missing and imputed (to country modal value) |
| Imputed:  Ability grouping (Different classes) | Binary = 1 if school-level variable identifying the presence of ability grouping in different classes is missing and imputed (to country modal value) |
| Imputed:  Ability grouping (Within classes) | Binary = 1 if school-level variable identifying the presence of ability grouping within classes is missing and imputed (to country modal value) |
| Imputed:  Lack Parents | Binary = 1 if individual-level variable signaling lack of parents is missing and imputed (to country modal value) |
| Imputed:  Bullying | Binary = 1 if any of the three questionnaire items concerning bullying used to construct the continuous measure of bullying used for the analysis is missing (missing values in each of the bullying-related questionnaire items are imputed to country modal values) |
| Imputed:  Perc. Competition | Binary = 1 if at least one student answer was imputed at the school level (this variable takes value 1 for 69.90% of the observations in the sample). |
| Imputed:  Perc. Cooperation | Binary = 1 if at least one student answer was imputed at the school level (this variable takes value 1 for 77.46% of the observations in the sample). |

# (A3). Descriptive statistics: School-level data

## Table A3: Number of schools by country (N=4819)

--------------------------------------------------------------------------------------------

| Country Freq. Percent Cum.  ----------------------------------------------- ---------   \| BEL \| 259 \| 5.37 \| 5.37 \| \| --- \| --- \| --- \| --- \| \| BGR \| 177 \| 3.67 \| 9.05 \| \| CZE \| 286 \| 5.93 \| 14.98 \| \| DEU \| 185 \| 3.84 \| 18.82 \| \| DNK \| 319 \| 6.62 \| 25.44 \| \| EST \| 182 \| 3.78 \| 29.22 \| \| FIN \| 199 \| 4.13 \| 33.35 \| \| FRA \| 225 \| 4.67 \| 38.02 \| \| GRC \| 201 \| 4.17 \| 42.19 \| \| HRV \| 179 \| 3.71 \| 45.90 \| \| HUN \| 161 \| 3.34 \| 49.24 \| \| IRL \| 157 \| 3.26 \| 52.50 \|   ----------------------------------------------------  Total 4819 100.00 | --------------------------------------------------------   \| ITA \| 460 \| 9.55 \| 62.05 \| \| --- \| --- \| --- \| --- \| \| LTU \| 256 \| 5.31 \| 67.36 \| \| LUX \| 41 \| 0.85 \| 68.21 \| \| LVA \| 243 \| 5.04 \| 73.25 \| \| MLT \| 48 \| 1.00 \| 74.25 \| \| NLD \| 148 \| 3.07 \| 77.32 \| \| POL \| 218 \| 4.52 \| 81.84 \| \| PRT \| 248 \| 5.15 \| 86.99 \| \| ROU \| 138 \| 2.86 \| 89.85 \| \| SVK \| 241 \| 5.00 \| 94.85 \| \| SVN \| 248 \| 5.15 \| 100.00 \| |
| --- | --- | --- | --- | --- | --- | --- | --- | --- | --- | --- | --- | --- | --- | --- | --- | --- | --- | --- | --- | --- | --- | --- | --- | --- | --- | --- | --- | --- | --- | --- | --- | --- | --- | --- | --- | --- | --- | --- | --- | --- | --- | --- | --- | --- | --- | --- | --- | --- | --- | --- | --- | --- | --- | --- | --- | --- | --- | --- | --- | --- | --- | --- | --- | --- | --- | --- | --- | --- | --- | --- | --- | --- | --- | --- | --- | --- | --- | --- | --- | --- | --- | --- | --- | --- | --- | --- | --- | --- | --- | --- | --- | --- | --- |

# (A4). Additional results from multilevel regressions

## Table A4.1: Extraction of multilevel regression results showing estimated coefficients

|  | VARIABLES | | (1)  Null model | (2)  Schools | (3)  + Individuals experiences in schools | (4)  + Individual characteristics | (5)  + Mental Health variables |
| --- | --- | --- | --- | --- | --- | --- | --- |
|  | Urban area |  | | 0.161*** | 0.201*** | 0.206*** | 0.178*** |
| **School characteristics** |  |  | | (0.055) | (0.057) | (0.057) | (0.058) |
|  | Imputation Dummy [Urban area] |  | | -0.027 | -0.027 | -0.019 | -0.003 |
|  |  |  | | (0.148) | (0.154) | (0.155) | (0.177) |
|  | School size§ |  | | -0.031 | -0.032 | -0.028 | -0.023 |
|  |  |  | | (0.022) | (0.023) | (0.023) | (0.023) |
|  | Imputation Dummy [School size§] |  | | 0.081 | 0.087 | 0.079 | 0.060 |
|  |  |  | | (0.093) | (0.096) | (0.095) | (0.105) |
|  | Share of Low SES |  | | -0.295*** | -0.252** | -0.254** | -0.216** |
|  |  |  | | (0.093) | (0.098) | (0.106) | (0.109) |
|  | Share of Migrant |  | | 0.122 | 0.120 | -0.024 | 0.016 |
|  |  |  | | (0.139) | (0.144) | (0.162) | (0.176) |
|  | Share of Repeating grade |  | | 0.122 | -0.097 | -0.120 | -0.047 |
|  |  |  | | (0.150) | (0.172) | (0.171) | (0.190) |
|  | Share of Lack Teachers |  | | 0.353*** | -0.073 | -0.063 | -0.107 |
|  |  |  | | (0.120) | (0.132) | (0.132) | (0.138) |
|  | Share of LOW Reading |  | | 0.289 | -0.011 | 0.078 | 0.053 |
|  |  |  | | (0.211) | (0.233) | (0.233) | (0.243) |
|  | Share of LOW Reading^2 |  | | 0.728*** | 0.684*** | 0.585** | 0.576** |
|  |  |  | | (0.243) | (0.258) | (0.258) | (0.268) |
|  | Perceived Competition |  | | 0.073 | 0.039 | 0.114 | 0.069 |
|  |  |  | | (0.120) | (0.124) | (0.125) | (0.131) |
|  | Imputation Dummy [Perc. Comp.] |  | | 0.031 | -0.003 | -0.011 | -0.007 |
|  |  |  | | (0.040) | (0.042) | (0.042) | (0.044) |
|  | Perceived Cooperation |  | | -0.666*** | -0.672*** | -0.626*** | -0.621*** |
|  |  |  | | (0.116) | (0.120) | (0.120) | (0.127) |
|  | Imputation Dummy [Perc. Coop.] |  | | 0.061 | 0.043 | 0.042 | 0.028 |
|  |  |  | | (0.043) | (0.045) | (0.045) | (0.048) |
|  | Bullying exp: moderate |  | | 0.310*** | 0.101** | 0.104** | 0.067 |
|  |  |  | | (0.041) | (0.043) | (0.043) | (0.045) |
|  | Bullying exp: high |  | | 0.617*** | 0.158*** | 0.160*** | 0.151*** |
|  |  |  | | (0.048) | (0.052) | (0.052) | (0.054) |
|  | Ability grouping: Diff. classes |  | | 0.034 | 0.038 | 0.036 | 0.026 |
|  |  |  | | (0.032) | (0.033) | (0.033) | (0.035) |
|  | Imputation Dummy [Ab. Gr: Diff.] |  | | -0.172* | -0.160 | -0.160 | -0.133 |
|  |  |  | | (0.098) | (0.102) | (0.101) | (0.104) |
|  | Ability grouping: Within classes |  | | 0.041 | 0.042 | 0.040 | 0.040 |
|  |  |  | | (0.031) | (0.032) | (0.032) | (0.033) |
|  | Imputation Dummy [Ab. Gr: With.] |  | | 0.014 | -0.019 | -0.018 | -0.056 |
|  |  |  | | (0.101) | (0.106) | (0.107) | (0.121) |
|  | Low n. school activities |  | | -0.036 | -0.033 | -0.029 | -0.035 |
|  |  |  | | (0.035) | (0.037) | (0.037) | (0.039) |
| **Experiences in school** | Repeat grade |  | |  | 0.183** | 0.178** | 0.133 |
|  |  |  | |  | (0.073) | (0.073) | (0.081) |
|  | TOP Reading group |  | |  | 0.141** | 0.134* | 0.103 |
|  |  |  | |  | (0.071) | (0.071) | (0.077) |
|  | LOW Reading group |  | |  | 0.266*** | 0.252*** | 0.278*** |
|  |  |  | |  | (0.056) | (0.056) | (0.059) |
|  | Bullying exp: moderate |  | |  | 0.998*** | 0.984*** | 0.921*** |
|  |  |  | |  | (0.041) | (0.042) | (0.044) |
|  | Bullying exp: high |  | |  | 1.976*** | 1.956*** | 1.756*** |
|  |  |  | |  | (0.059) | (0.060) | (0.063) |
|  | Imputation Dummy [Bullying] |  | |  | 0.617*** | 0.482*** | 0.472*** |
|  |  |  | |  | (0.059) | (0.077) | (0.082) |
|  | Lack Teachers |  | |  | 0.399*** | 0.358*** | 0.290*** |
|  |  |  | |  | (0.038) | (0.039) | (0.041) |
| **Individual characteristics** | Female |  | |  |  | 0.263*** | 0.131*** |
|  |  |  | |  |  | (0.037) | (0.039) |
|  | Migrant (1st gen) |  | |  |  | 0.371*** | 0.455*** |
|  |  |  | |  |  | (0.120) | (0.129) |
|  | Migrant (2nd gen) |  | |  |  | -0.141 | -0.112 |
|  |  |  | |  |  | (0.092) | (0.095) |
|  | Diff. Language |  | |  |  | 0.129* | 0.084 |
|  |  |  | |  |  | (0.076) | (0.083) |
|  | Low SES |  | |  |  | -0.009 | -0.025 |
|  |  |  | |  |  | (0.041) | (0.043) |
|  | Lack Parents |  | |  |  | 0.550*** | 0.412*** |
|  |  |  | |  |  | (0.046) | (0.049) |
|  | Imputation Dummy [Lack Parents] |  | |  |  | 0.307*** | 0.274*** |
|  |  |  | |  |  | (0.095) | (0.099) |
| **Mental health** | Feel afraid |  | |  |  |  | 0.485*** |
|  |  |  | |  |  |  | (0.065) |
|  | Feel sad |  | |  |  |  | 1.055*** |
|  |  |  | |  |  |  | (0.069) |
|  | Lack belief |  | |  |  |  | 0.842*** |
|  |  |  | |  |  |  | (0.069) |
|  | Imputation Dummy [Lack belief] |  | |  |  |  | 0.040 |
|  |  |  | |  |  |  | (0.175) |
|  | *Constant* | -1.958***  (0.078) | | -2.439***  (0.157) | -2.981***  (0.164) | -3.253*** (0.167) | -3.117*** (0.177) |
|  | Country F.E.  N. Observations  N. Schools  Log-Likelihood  VPC/ICC  Sigma u | Yes  118.698  4.819  -991432  0.229  0.979***  (0.052) | | Yes  118.698  4.819  -991130  0.201  0.827***  (0.047) | Yes  118.698  4.819  -926129  0.214  0.893***  (0.049) | Yes  118.698  4.819  -917354  0.213  0.889***  (0.049) | Yes  111.567  4.560  -860906  0.216  0.906***  (0.052) |

Source: PISA 2018, authors’ calculations.

Note: The table shows estimated coefficients based on multi-level analyses using PISA weights. The analyses are based on 118698students in 4819 schools in 23 countries. Country fixed effects are used, baseline country is Romania, where the country-average share of lonely students is 14.7% (.35 sd), narrowly comparable to the average share of lonely students in the pooled sample, equal to 14.6%, (.35 sd). Significance levels: * denotes p<0.10, ** denotes p<0.05 and *** p<0.01. Variables marked with ‘§’ are standardized within the pooled sample with school-level data on the 23 EU countries in the analysis.

## Table A4.2.a: Extraction of multilevel regression results with interactions (est. coeff)

|  | VARIABLES | | (1) | (2) | (3) | (4) |
| --- | --- | --- | --- | --- | --- | --- |
|  | Urban area | 0.202*** | | 0.198*** | 0.205*** | 0.205*** |
| **School characteristics** |  | (0.057) | | (0.057) | (0.057) | (0.056) |
|  | Imputation Dummy [Urban area] | -0.014 | | -0.015 | -0.018 | -0.015 |
|  |  | (0.155) | | (0.155) | (0.155) | (0.155) |
|  | School size§ | -0.029 | | -0.028 | -0.028 | -0.029 |
|  |  | (0.023) | | (0.023) | (0.023) | (0.023) |
|  | Imputation Dummy [School size§] | 0.075 | | 0.078 | 0.078 | 0.079 |
|  |  | (0.095) | | (0.096) | (0.095) | (0.095) |
|  | Share of Low SES | -0.250** | | -0.011 | -0.253** | -0.272** |
|  |  | (0.106) | | (0.135) | (0.106) | (0.106) |
|  | Share of Migrant | 0.219 | | -0.033 | -0.023 | -0.021 |
|  |  | (0.210) | | (0.161) | (0.161) | (0.161) |
|  | Share of Repeating grade | -0.111 | | -0.123 | -0.120 | -0.136 |
|  |  | (0.171) | | (0.171) | (0.171) | (0.172) |
|  | Share of Lack Teachers | -0.071 | | -0.066 | -0.064 | -0.065 |
|  |  | (0.132) | | (0.132) | (0.132) | (0.132) |
|  | Share of LOW Reading | 0.063 | | 0.044 | -0.055 | 0.392 |
|  |  | (0.233) | | (0.234) | (0.244) | (0.371) |
|  | Share of LOW Reading^2 | 0.603** | | 0.639** | 1.035*** | 0.257 |
|  |  | (0.259) | | (0.260) | (0.373) | (0.598) |
|  | Perceived Competition | 0.120 | | 0.119 | 0.114 | 0.109 |
|  |  | (0.125) | | (0.125) | (0.125) | (0.125) |
|  | Imputation Dummy [Perc. Comp.] | -0.013 | | -0.010 | -0.012 | -0.011 |
|  |  | (0.042) | | (0.042) | (0.042) | (0.042) |
|  | Perceived Cooperation | -0.623*** | | -0.616*** | -0.625*** | -0.616*** |
|  |  | (0.120) | | (0.120) | (0.120) | (0.120) |
|  | Imputation Dummy [Perc. Coop.] | 0.041 | | 0.039 | 0.042 | 0.040 |
|  |  | (0.045) | | (0.045) | (0.045) | (0.045) |
|  | Bullying exp: moderate | 0.101** | | 0.104** | 0.104** | 0.101** |
|  |  | (0.043) | | (0.043) | (0.043) | (0.042) |
|  | Bullying exp: high | 0.157*** | | 0.159*** | 0.160*** | 0.158*** |
|  |  | (0.053) | | (0.053) | (0.052) | (0.052) |
|  | Ability grouping: Diff. classes | 0.036 | | 0.035 | 0.036 | 0.037 |
|  |  | (0.034) | | (0.033) | (0.033) | (0.033) |
|  | Imputation Dummy [Ab. Gr: Diff.] | -0.162 | | -0.162 | -0.161 | -0.164 |
|  |  | (0.101) | | (0.101) | (0.101) | (0.101) |
|  | Ability grouping: Within classes | 0.040 | | 0.038 | 0.040 | 0.039 |
|  |  | (0.032) | | (0.032) | (0.032) | (0.032) |
|  | Imputation Dummy [Ab. Gr: With.] | -0.019 | | -0.021 | -0.019 | -0.021 |
|  |  | (0.107) | | (0.107) | (0.107) | (0.107) |
|  | Low n. school activities | -0.030 | | -0.028 | -0.029 | -0.030 |
|  |  | (0.037) | | (0.037) | (0.037) | (0.037) |
| **Experiences in school** | Repeat grade | 0.173** | | 0.178** | 0.178** | 0.179** |
|  |  | (0.073) | | (0.073) | (0.073) | (0.073) |
|  | TOP Reading group | 0.134* | | 0.139* | 0.138* | 0.141** |
|  |  | (0.071) | | (0.071) | (0.072) | (0.072) |
|  | LOW Reading group | 0.254*** | | 0.250*** | 0.393*** | 0.554*** |
|  |  | (0.056) | | (0.056) | (0.098) | (0.150) |
|  | Bullying exp: moderate | 0.983*** | | 0.984*** | 0.985*** | 0.985*** |
|  |  | (0.042) | | (0.042) | (0.042) | (0.018) |
|  | Bullying exp: high | 1.955*** | | 1.955*** | 1.956*** | 1.956*** |
|  |  | (0.060) | | (0.060) | (0.060) | (0.056) |
|  | Imputation Dummy [Bullying] | 0.484*** | | 0.481*** | 0.484*** | 0.484*** |
|  |  | (0.078) | | (0.077) | (0.077) | (0.077) |
|  | Lack Teachers | 0.357*** | | 0.357*** | 0.358*** | 0.358*** |
|  |  | (0.039) | | (0.039) | (0.039) | (0.039) |
| **Individual characteristics** | Female | 0.263*** | | 0.263*** | 0.264*** | 0.265*** |
|  |  | (0.037) | | (0.037) | (0.037) | (0.037) |
|  | Migrant (1st gen) | 0.548*** | | 0.366*** | 0.370*** | 0.370*** |
|  |  | (0.144) | | (0.120) | (0.119) | (0.120) |
|  | Migrant (2nd gen) | 0.044 | | -0.148 | -0.142 | -0.143 |
|  |  | (0.131) | | (0.092) | (0.092) | (0.092) |
|  | Diff. Language | 0.130* | | 0.128* | 0.129* | 0.130* |
|  |  | (0.076) | | (0.076) | (0.076) | (0.076) |
|  | Low SES | -0.008 | | 0.248** | -0.011 | -0.011 |
|  |  | (0.041) | | (0.108) | (0.041) | (0.041) |
|  | Lack Parents | 0.551*** | | 0.548*** | 0.551*** | 0.551*** |
|  |  | (0.046) | | (0.046) | (0.046) | (0.046) |
|  | Imputation Dummy [Lack Parents] | 0.308*** | | 0.307*** | 0.306*** | 0.306*** |
|  |  | (0.095) | | (0.095) | (0.095) | (0.094) |
| **Interactions** | Migr x Share Migr | -0.742* | |  |  |  |
|  |  | (0.399) | |  |  |  |
|  | LowSES x Share LowSES |  | | -0.557*** |  |  |
|  |  |  | | (0.207) |  |  |
|  | LOWRead x Share LOWRead |  | |  | -0.446* | -1.673* |
|  |  |  | |  | (0.269) | (0.870) |
|  | LOWRead x Share LOWRead^2 |  | |  |  | 1.596 |
|  |  |  | |  |  | (1.027) |
|  | *Constant* | -3.252***  (0.167) | | -3.343***  (0.170) | -3.257***  (0.167) | -3.269***  (0.165) |
|  | Country F.E.  N. Observations  N. Schools  Log-Likelihood  VPC/ICC  Sigma u | Yes  118.698  4.819  -917179  0.213  0.891***  (0.049) | | Yes  118.698  4.819  -917060  0.213  0.890***  (0.049) | Yes  118.698  4.819  -917224  0.213  0.889***  (0.049) | Yes  118.698  4.819  -917139  0.213  0.889***  (0.049) |

Source: PISA 2018, authors’ calculations.

Note: The table shows estimated coefficients based on multi-level analyses using PISA weights. The analyses are based on 118698students in 4819 schools in 23 countries. Country fixed effects are used, baseline country is Romania, where the country-average share of lonely students is 14.7% (.35 sd), narrowly comparable to the average share of lonely students in the pooled sample, equal to 14.6%, (.35 sd). Significance levels: * denotes p<0.10, ** denotes p<0.05 and *** p<0.01. Variables marked with ‘§’ are standardized within the pooled sample with school-level data on the 23 EU countries in the analysis.

## Table A4.2.a: Extraction of multilevel regression results with interactions (est. coeff)

|  | VARIABLES | | (1) | (2) | (3) | (4) |
| --- | --- | --- | --- | --- | --- | --- |
|  | Urban area | 0.207*** | | 0.206*** | 0.204*** | 0.207*** |
| **School characteristics** |  | (0.057) | | (0.057) | (0.056) | (0.057) |
|  | Imputation Dummy [Urban area] | -0.017 | | -0.017 | -0.018 | -0.020 |
|  |  | (0.155) | | (0.155) | (0.155) | (0.155) |
|  | School size§ | -0.029 | | -0.029 | -0.028 | -0.028 |
|  |  | (0.023) | | (0.023) | (0.023) | (0.023) |
|  | Imputation Dummy [School size§] | 0.078 | | 0.078 | 0.082 | 0.080 |
|  |  | (0.095) | | (0.095) | (0.096) | (0.095) |
|  | Share of Low SES | -0.261** | | -0.266** | -0.254** | -0.255** |
|  |  | (0.106) | | (0.106) | (0.106) | (0.106) |
|  | Share of Migrant | -0.024 | | -0.022 | -0.025 | -0.022 |
|  |  | (0.162) | | (0.162) | (0.162) | (0.161) |
|  | Share of Repeating grade | -0.121 | | -0.122 | -0.110 | -0.120 |
|  |  | (0.171) | | (0.171) | (0.171) | (0.171) |
|  | Share of Lack Teachers | -0.065 | | -0.066 | -0.064 | -0.064 |
|  |  | (0.132) | | (0.132) | (0.132) | (0.132) |
|  | Share of LOW Reading | 0.016 | | 0.004 | 0.074 | 0.084 |
|  |  | (0.237) | | (0.237) | (0.233) | (0.233) |
|  | Share of LOW Reading^2 | 0.648** | | 0.666** | 0.589** | 0.580** |
|  |  | (0.261) | | (0.262) | (0.258) | (0.258) |
|  | Perceived Competition | 0.115 | | 0.115 | 0.116 | 0.323 |
|  |  | (0.125) | | (0.125) | (0.125) | (0.347) |
|  | Imputation Dummy [Perc. Comp.] | -0.011 | | -0.011 | -0.012 | -0.011 |
|  |  | (0.042) | | (0.042) | (0.042) | (0.042) |
|  | Perceived Cooperation | -0.624*** | | -0.622*** | -0.621*** | -0.496** |
|  |  | (0.120) | | (0.120) | (0.120) | (0.250) |
|  | Imputation Dummy [Perc. Coop.] | 0.041 | | 0.041 | 0.039 | 0.043 |
|  |  | (0.045) | | (0.045) | (0.045) | (0.045) |
|  | Bullying exp: moderate | 0.103** | | 0.103** | 0.092* | 0.104** |
|  |  | (0.043) | | (0.043) | (0.054) | (0.043) |
|  | Bullying exp: high | 0.160*** | | 0.160*** | 0.265*** | 0.158*** |
|  |  | (0.053) | | (0.052) | (0.057) | (0.053) |
|  | Ability grouping: Diff. classes | 0.036 | | 0.036 | 0.036 | 0.037 |
|  |  | (0.033) | | (0.033) | (0.034) | (0.033) |
|  | Imputation Dummy [Ab. Gr: Diff.] | -0.160 | | -0.160 | -0.162 | -0.160 |
|  |  | (0.101) | | (0.101) | (0.101) | (0.101) |
|  | Ability grouping: Within classes | 0.039 | | 0.039 | 0.040 | 0.039 |
|  |  | (0.032) | | (0.032) | (0.032) | (0.032) |
|  | Imputation Dummy [Ab. Gr: With.] | -0.019 | | -0.019 | -0.023 | -0.018 |
|  |  | (0.107) | | (0.107) | (0.107) | (0.107) |
|  | Low n. school activities | -0.029 | | -0.029 | -0.028 | -0.029 |
|  |  | (0.037) | | (0.037) | (0.037) | (0.037) |
| **Experiences in school** | Repeat grade | 0.179** | | 0.178** | 0.173** | 0.178** |
|  |  | (0.073) | | (0.073) | (0.073) | (0.073) |
|  | TOP Reading group | 0.066 | | 0.028 | 0.135* | 0.134* |
|  |  | (0.084) | | (0.091) | (0.071) | (0.071) |
|  | LOW Reading group | 0.257*** | | 0.258*** | 0.255*** | 0.252*** |
|  |  | (0.056) | | (0.056) | (0.056) | (0.056) |
|  | Bullying exp: moderate | 0.984*** | | 0.984*** | 0.985*** | 0.984*** |
|  |  | (0.042) | | (0.042) | (0.066) | (0.042) |
|  | Bullying exp: high | 1.957*** | | 1.956*** | 2.148*** | 1.956*** |
|  |  | (0.060) | | (0.060) | (0.074) | (0.060) |
|  | Imputation Dummy [Bullying] | 0.482*** | | 0.482*** | 0.485*** | 0.482*** |
|  |  | (0.078) | | (0.078) | (0.077) | (0.077) |
|  | Lack Teachers | 0.358*** | | 0.358*** | 0.357*** | 0.358*** |
|  |  | (0.039) | | (0.039) | (0.039) | (0.039) |
| **Individual characteristics** | Female | 0.262*** | | 0.262*** | 0.261*** | 0.263*** |
|  |  | (0.037) | | (0.037) | (0.037) | (0.037) |
|  | Migrant (1st gen) | 0.371*** | | 0.372*** | 0.364*** | 0.371*** |
|  |  | (0.120) | | (0.120) | (0.120) | (0.120) |
|  | Migrant (2nd gen) | -0.141 | | -0.140 | -0.141 | -0.141 |
|  |  | (0.092) | | (0.092) | (0.092) | (0.092) |
|  | Diff. Language | 0.129* | | 0.129* | 0.130* | 0.129* |
|  |  | (0.076) | | (0.076) | (0.075) | (0.076) |
|  | Low SES | -0.008 | | -0.008 | -0.010 | -0.009 |
|  |  | (0.041) | | (0.041) | (0.041) | (0.041) |
|  | Lack Parents | 0.550*** | | 0.549*** | 0.546*** | 0.550*** |
|  |  | (0.046) | | (0.046) | (0.046) | (0.046) |
|  | Imputation Dummy [Lack Parents] | 0.306*** | | 0.306*** | 0.307*** | 0.307*** |
|  |  | (0.095) | | (0.095) | (0.095) | (0.095) |
| **Interactions** | Migr x Share Migr | 1.350* | | 3.051* |  |  |
|  |  | (0.792) | | (1.630) |  |  |
|  | LowSES x Share LowSES |  | | -5.909 |  |  |
|  |  |  | | (4.324) |  |  |
|  | LOWRead x Share LOWRead |  | |  | -0.019 |  |
|  |  |  | |  | (0.083) |  |
|  | LOWRead x Share LOWRead^2 |  | |  | -0.536*** |  |
|  |  |  | |  | (0.115) |  |
|  | School PercCOOP x School PercCOMP |  | |  |  | -0.333 |
|  |  |  | |  |  | (0.525) |
|  | *Constant* | -3.243*** (0.167) | | -3.240***  (0.167) | -3.255***  (0.168) | -3.327***  (0.208) |
|  | Country F.E.  N. Observations  N. Schools  Log-Likelihood  VPC/ICC  Sigma u | Yes  118.698  4.819  -917265  0.213  0.889***  (0.049) | | Yes  118.698  4.819  -917218  0.213  0.889***  (0.049) | Yes  118.698  4.819  -916293  0.213  0.892***  (0.049) | Yes  118.698  4.819  -917354  0.213  0.889***  (0.049) |

1. At this stage, after the imputation procedure, we still observe missing values for 7163 observations from Belgium: we have no non-missing observation about students’ self-reporting to feel “Sad” or “Afraid” at the country level hence the imputation procedure, based on country modal values, fails. For this reason, we only include these variables in secondary analyses and refrain from dropping all missing observations, which would result in excluding Belgium from the list of countries included in the study. [↑](#footnote-ref-1)
2. ibidem [↑](#footnote-ref-2)
3. Since imputation relies on country averages and modes, the presence of missing values at this stage implies the variable is missing for all the observations from a certain country. As a result, applying this restriction criterion causes the exclusion of two additional countries from the sample: Austria is excluded because no information on interviewed students’ schools size is available; Sweden is excluded because no information on interviewed students’ school location (rural vs. urban) is available. [↑](#footnote-ref-3)
